# The negative effects of dry eye disease on quality of life and visual function

**DOI:** 10.3906/sag-2002-143

**Published:** 2020-11-03

**Authors:** Lee Guo W. O.D, Esen K AKPEK

**Affiliations:** 1 Department of Ocular Surface Diseases and Dry Eye Clinic, The Wilmer Eye Institute, Johns Hopkins University, Baltimore, Maryland USA

**Keywords:** Dry eye, quality of life, visual function, functional visual acuity, patient questionnaire, OSDI, SPEED, SANDE, IDEEL, NEI VFQ-25

## Abstract

In recent years, dry eye has become a hot topic within ophthalmology and optometry, especially in regards to new frontiers in treatment modalities which include novel devices, procedures, and medications. However, some of the more understudied areas in dry eye involve its impact on quality of life. Although ocular discomfort symptoms are well known to be associated with dry eye, its negative effects on visual function remain underrecognized. This paper reviews these topics within the currently published literature to heighten awareness among clinicians. **Key Words: D** ry eye, quality of life, visual function, functional visual acuity, patient questionnaire, OSDI, SPEED, SANDE, IDEEL, NEI VFQ-25

## 1. Introduction

Among all ocular conditions, dry eye remains one of the most prevalent in clinical practice, ranging from 5% to 50% with higher variations in signs than symptoms [1]. Increased age, female sex, and Asian ethnicity are among the most consistent risk factors for developing dry eye disease [1]. In fact, the various signs and symptoms of dry eye often prompt patients to seek eye care due to its widespread affect on day to day life. 

Currently, the most broadly accepted definition of dry eye disease revolves around the Tear Film and Ocular Surface Society International Dry Eye Workshop (TFOS DEWS II) in 2017 as a “multifactorial disease of the ocular surface characterized by a loss of homeostasis of the tear film, and accompanied by ocular symptoms, in which tear film instability and hyperosmolarity, ocular surface inflammation and damage, and neurosensory abnormalities play etiological roles.” [1] The vast complexities of pathophysiology, immunology, epidemiology, genetics, mechanisms of action, diagnosis, and treatment/management approaches continue to be extensively investigated and researched worldwide. We will herein emphasize the negative effects of dry eye on quality of life, particularly as it relates to visual symptoms commonly reported by the afflicted patients.

## 2. Dry eye and quality of life

The World Health Organization recognizes quality of life to be a multifaceted concept incorporating how health conditions can alter (1) physical health, (2) psychological wellbeing, (3) level of independence, (4) environmental impact, (5) social relationships, and (6) spirituality/religion/personal beliefs [2]. 

Among these quality of life categories, dry eye most directly affects the first four [2]:

### 2.1. Physical health 

- **Pain and discomfort:** Dry eye patients will frequently complain of varying forms of ocular pain and discomfort including sharpness, dullness, stinging, burning, pressure sensation, throbbing, foreign body sensation, sandiness, grittiness, redness, tearing/watering, stringy mucous discharge, eye strain, eye fatigue, heavy eyelids, contact lens intolerance, and light sensitivity [3,4,8].

- **Blurred vision** : Inadequate tear production and/or poor quality tears characteristic to dry eye disease can cause intermittent blurred or fluctuating vision. In addition, glare or haloes around lights at night [3] can also result due to poor tear film quality.

- **Poor sleep quality** : A 2016 study implementing the Pittsburgh sleep quality index found that among 672 participants ages 26–64, poor sleep quality was associated with dry eye disease[5].

### 2.2. Psychological 

- **Bodily image, appearance, self esteem, negative feelings:** Dry eye is often associated with chronic red hyperemic eyes, especially those suffering from redness associated with ocular rosacea[6]. Physical appearance of redness can negatively influence emotional health leading to comorbid anxiety disorders and social phobias[6]. In fact, a 2016 large population systemic review and meta-analysis study found that depression and anxiety are more prevalent among patients suffering from dry eye disease, specifically those dry eye patients afflicted with primary Sjögren’s syndrome who were found to have the highest prevalence and severity of depression[7].

- **Thinking, learning, memory, concentration:** Studies on chronic pain syndromes similar to dry eye might explain how it can negatively affect cognitive processes, sleep, mood, and mental health [4,7]. Chronic dry eye also causes fluctuating vision which can slow reading rates and significantly disrupt day to day tasks that require visual concentration for long periods of time [8]. 

### 2.3. Level of independence

- **Activities of daily living:** Because dry eye is a chronic incurable condition, both palliative and medicinal management can be lifelong, creating the added burden of integrating dry eye treatment regimens with activities of daily living. Studies support that patients who are older, have poor vision, or have limited schooling struggle the most with adhering to dry eye treatment compliance [9–12]. Furthermore, patients with poor manual dexterity secondary to neurodegenerative and autoimmune pathology (dementia [11], Parkinson’s, multiple sclerosis, rheumatoid arthritis, etc) may also experience limited independence from being unable to handle eye drop instillations themselves.

- **Dependence on medicinal substances and medical aids:** Longterm management of dry eye symptomatology includes topical immunomodulators, topical corticosteroids, artificial tears/gels/ointments, eyelid/eyelash cleansers, punctal plugs, omega-3 fatty acid supplements, antibiotics (topical and systemic), moisture goggles, and autologous serum tears [4].

- **Work capacity:** Many published works support dry eye disease to have negative effects on concentration [7,8] and decreased productivity [1,13,14]. More specifically, studies found dry eye to reduce workplace [13,14] and nonjob related [13] performances and create substantial loss to work industry [14].

### 2.4. Environmental impact

- **Financial resources:** The economic burden of long-term dry eye management can be significant, including the costs of medical eye office visits, medications, and dietary or palliative supplements. In fact, a 2011 survey study among 2,171 dry eye patients found the average annual cost to be $783 with an overall burden of dry eye disease for the U.S. healthcare system to be an estimated $3.84 billion [15]. Furthermore, costs are higher among patients suffering from moderate to severe forms of dry eye disease [15].

- **Participation in and opportunities for recreation/** **leisure:** Dry eye disease can negatively affect nonjob related [13] activities including sports where fluctuating visual acuity due to poor tear film dynamics can compromise one’s accuracy of visual tracking and fixation ability on moving targets whilst incorporating hand eye coordination.

- **Physical environment (pollution/noise/traffic/climate):** Multiple external and environmental risk factors can exacerbate dry eye disease, especially air pollutants. More specifically, O3, PM2.5, and SO2 were associated with dry eye disease according to a 2019 large population study in China[16].

## 3. Quality of life assessment and patient questionnaires

In recent years, various self-reported outcome measure questionnaires have been created to assess dry eye symptoms and how they impact various aspects of quality of life. We will review five major questionnaires commonly utilized across eye clinics and academic centers as primary means to assess symptomatology and effects on quality of life. 

### 3.1. Ocular surface disease index (OSDI):

Twelve patient response items in a points summation system assesses symptoms, functional limitations, and environmental factors related to dry eye[17,18]. Three subsections account for vision-related function, dry eye symptoms, and environmental triggers[18]. The OSDI is among the most commonly utilized survey questionnaires utilized to assess ocular surface disease severity in dry eye research[17,18] including patients with Sjögren’s syndrome[19], glaucoma [20], and Graves’ disease[21]. Most importantly, the OSDI has been implemented to assess the efficacy of various dry eye treatments[22–24]. One major drawback to the OSDI is its limited account on the effects of dry eye disease on vision-related functioning as opposed to severity alone; therefore it is less likely to describe the full impact and burden of dry eye on everyday life compared to other comprehensive questionnaires like the impact of dry eye on everyday life (IDEEL) [25] and the National Eye Institute’s visual function questionnaire **(** NEI VFQ-25). Overall however, OSDI has proven to be a valuable patient reported outcome measure in ophthalmology research and clinical trials[26].

### 3.2. Standard patient evaluation of eye dryness (SPEED):

Similar to OSDI, SPEED also utilizes twelve patient response items in a points summation system. However unlike OSDI, the three subsections in SPEED solely focus on symptoms: type, frequency, and severity. Studies found while both OSDI and SPEED are suitable for detecting dry eye symptoms, their results cannot be used interchangeably because SPEED correlated more with evaporative dry eye while OSDI correlated more with aqueous tear-deficient dry eye [27]. Certain studies support SPEED being superior to OSDI in differentiating between asymptomatic vs symptomatic patients [28]. Overall, SPEED has been shown to be repeatable and valid instrument for measuring dry eye symptoms correlating significantly with ocular surface staining and clinical measures of Meibomian gland dysfunction [29].

### 3.3. Symptom assessment questionnaire in dry eye (SANDE):

Unlike OSDI, the SANDE is a more simplified questionnaire utilizing a two-questioned 100 mm horizontal linear visual analog scale, quantifying both severity and frequency of dry eye symptoms. A 2015 study demonstrated that SANDE had significant correlation and negligible score differences to OSDI implying its potential to provide shorter, quicker, and comparably reliable measures of dry eye symptoms to that of OSDI [30]. Recent 2019 studies found that both OSDI and SANDE have superior discriminative ability in detecting dry eye signs compared to other questionnaires including SPEED [31]. SANDE has also been utilized to assess the efficacy of certain dry eye treatments including the new TearCare system [32]. While its simplicity certainly improves efficiency in everyday clinical practice for general dry eye screening purposes, SANDE’s less detailed nature limits its ability to cover the breadth of information required to better assess dry eye effects on quality of life.

### 3.4. Impact of dry eye on everyday life (IDEEL):

Unlike other questionnaires, the IDEEL is lengthy and more comprehensive, involving three modules and 57 questions that assess (1) dry eye symptoms and severity, (2) dry eye’s impact on quality of life including daily activities, emotional feelings, and work/productivity, and (3) treatment satisfaction [25,26]. Studies have found that IDEEL better discriminates dry eye severity levels than other quality of life scales [33]. While the main drawback of IDEEL is the glaring inefficiency (approximately 30 min to complete), it meets the new FDA patient reported outcome instrument guidelines and aligns with validity and reliability standards [25]. Furthermore, IDEEL contains the most quality of life measures of any current dry eye questionnaire [26].

### 3.5. National Eye Institute’s visual function questionnaire (NEI VFQ-25):

This questionnaire focuses on visual function impairments secondary not only to dry eye but other diseases including cataracts, macular degeneration, diabetic retinopathy, glaucoma, stroke-related vision loss, or low vision[34-41]. The 25-item approach includes several visual domains (general, distance, near, peripheral, driving, color), ocular pain scales, and five non-visual domains (general health, mental health, dependency, social function, role limitations) [9]. Overall, multiple studies continue to implement NEI VFQ-25 as a valid cross-sectional measure of how various diseases can impact visual function and quality of life.

## 4. Dry eye and visual function

Visual acuity represents the static measurement of image sharpness/resolution on a focal plane in an optical system (retina in the human eye). Visual function on the other hand, has more to do with the broader concept of how well visual acuity remains optimized in the setting of dynamic day-to-day tasks. Image quality to the retina is dependent on a clear light path through all ocular structures. The pre-corneal tear film is the first structure having direct influence on the optical light path [42]. When dry eye disease is present, the tear film is degraded often due to insufficient tear secretion quantity (aqueous tear deficiency) and/or poor tear film quality and stability leading to early break-up (evaporative Meibomian gland dysfunction-related lipid tear deficiency or inflammatory mucin tear deficiency) [43].

Functional visual acuity is a contrast measurement of visual acuity during and after sustained visual activity, which is argued to be more accurately representative of visual function in real-life situations like reading, computer work, and driving [9]. In fact, dry eye disease has been found in multiple studies to degrade visual function with sustained visual tasks like reading [8,44,45], digital device use (computer vision syndrome) [46], and driving [47]. Furthermore, studies support that dry eye disease negatively affects contrast sensitivity [48] and is associated with irregular astigmatism and higher order optical aberrations [49]. Dry eye causes increased straylight secondary to unstable tear film and reduces contrast sensitivity due to central superficial punctate keratopathy [50]. Therefore, a consistently stable tear film is critical to maintaining image quality in optimized visual functioning.

In conclusion, there continues to be growing evidence in the literature that dry eye disease negatively impacts quality of life and visual function across more domains than healthcare providers may realize. This may explain the paradigm shift in eye care now homing in on assessing visual function as opposed to ocular discomfort symptoms alone. While multiple questionnaires are currently available to quantify the effects of dry eye in clinical practice, dry eye diagnosis often remains missed during common clinical testing. Improved standards in patient care will remain dependent on doctors being more cognizant of how significantly tear film pathology can negatively affect their patient’s visual function day by day.
